# Age-related susceptibility to paraquat-induced neurotoxicity in male wistar rats: effects on neurobehaviour, substantia nigra cytoarchitecture, and alpha-synuclein levels

**DOI:** 10.1186/s12868-026-01019-0

**Published:** 2026-06-18

**Authors:** Kelechi Ichie, Azuoma Asomugha, Izuchukwu Okafor

**Affiliations:** 1 Department of Anatomy, Nnamdi Azikiwe University, Awka, Nigeria; 2 Nnamdi Azikiwe University Teaching Hospital, Nnewi, Nigeria; 3 Division of Translational Anatomy, Department of Radiology, University of Massachusetts T.H Chan Medical School, Worcester, USA

**Keywords:** Paraquat, Alpha-synuclein, Age-related susceptibility, Parkinsonism, Substantia nigra, Wistar rats

## Abstract

**Background:**

Parkinson’s disease (PD) is a progressive neurodegenerative disorder strongly associated with dopaminergic neuronal degeneration and alpha-synuclein pathology. Paraquat (PQ) has been implicated in Parkinsonian neurodegeneration; however, the influence of age on susceptibility to PQ-induced neuropathology remains insufficiently characterized.

**Aim:**

This study investigated age-dependent effects of paraquat exposure on neurobehaviour, substantia nigra histomorphology, and serum alpha-synuclein levels in male Wistar rats.

**Methods:**

Sixty-three male Wistar rats were assigned into juvenile, young adult, and adult age categories, each further subdivided into control, PQ-treated, and PQ+recovery groups. Paraquat (10 mg/kg, intraperitoneally) was administered twice weekly for three weeks. Recovery groups were maintained for a two-month post-exposure period. Neurobehavioral assessments were conducted to evaluate locomotor and anxiety-related functions. Serum and nigral alpha-synuclein concentrations were quantified using enzyme-linked immunosorbent assay (ELISA), while histological examination of the substantia nigra was performed to assess neuronal integrity.

**Results:**

Adult rats exhibited a significant reduction in locomotor activity following PQ exposure (*p* = 0.020) and showed more prominent histopathological alterations within the substantia nigra compared with juvenile and young-adult animals. Although improvement in tissue architecture was observed following paraquat withdrawal, residual alterations persisted, particularly in adults. Nigral alpha-synuclein concentrations did not differ significantly among treatment groups in any age cohort. Serum alpha-synuclein levels were similarly unchanged in most groups, except for a reduction observed in recovering young-adult animals (*p* = 0.039).

**Conclusion:**

Age influences vulnerability to PQ-induced neurotoxicity, with adult animals showing greater susceptibility to behavioral and histological damage. However, serum total alpha-synuclein levels did not consistently parallel central neuropathology, suggesting limited reliability as a standalone peripheral biomarker of PQ-induced Parkinsonism.

## Introduction

 Parkinsonian syndromes include a heterogeneous group of disorders characterized by dopaminergic dysfunction, of which idiopathic Parkinson’s disease (PD) represents the majority, while secondary Parkinsonism arises from environmental toxins, drugs, or metabolic insults [1, 2]. PD is mainly characterized by motor abnormalities including bradykinesia, resting tremor, muscular rigidity, and impaired postural balance. In addition, affected individuals frequently experience non-motor complications such as depression, sleep disturbances, autonomic dysfunction, sensory abnormalities, and cognitive decline [3–5]. These clinical manifestations largely arise from degeneration of dopaminergic neurons within the substantia nigra pars compacta (SNpc), resulting in reduced dopaminergic transmission to the striatum and subsequent impairment of motor coordination [3].

One of the principal neuropathological hallmarks of PD is the abnormal accumulation of alpha-synuclein (α-syn), a presynaptic neuronal protein involved in synaptic vesicle regulation [6–8]. Under pathological conditions, α-syn undergoes conformational misfolding and aggregates into oligomeric and fibrillary structures which disrupt neuronal homeostasis, promote oxidative injury, and stimulate neuroinflammatory pathways [9]. Furthermore, mutations and multiplications involving the synuclein alpha gene (SNCA) have been strongly implicated in familial forms of PD by enhancing α-syn aggregation and neuronal vulnerability [10].

Several biological and demographic variables influence the onset and progression of PD, among which age and sex remain particularly important [11]. Epidemiological evidence consistently demonstrates a higher incidence and severity in males than females, a phenomenon partly attributed to sex hormone–related differences in neuroprotection and α-syn regulation [12]. Nevertheless, aging remains the strongest known risk factor for PD. Advancing age is associated with mitochondrial dysfunction, impaired proteasomal activity, defective protein clearance mechanisms, and increased oxidative stress, all of which promote nigrostriatal neurodegeneration and favor α-syn accumulation [13].

Alongside genetic risk, environmental exposures substantially contribute to PD pathogenesis [14, 15]. Numerous toxicants—including heavy metals, pesticides, and neurotoxins such as rotenone, 6-Hydroxydopamine, MPTP, and paraquat—have been implicated in Parkinsonian degeneration [16, 17]. Paraquat (PQ) is especially notable because of its epidemiological association with PD and its ability to undergo redox cycling that generates reactive oxygen species (ROS), disrupts mitochondrial electron transport, and induces oxidative damage resembling idiopathic PD. These features explain its widespread use in experimental models of Parkinsonism [18, 19].

The detection of α-syn in peripheral biological fluids including cerebrospinal fluid (CSF), plasma, and serum has stimulated growing interest in its use as a minimally invasive biomarker for PD-related pathology [20–22]. While CSF analysis provides important neurochemical information, the invasive nature of sample collection limits its practicality for repeated monitoring and large-scale studies. In contrast, serum and plasma are more readily accessible and contain abundant α-syn derived largely from erythrocytes and platelets [23, 24]. Circulating α-syn measurements may therefore reflect both neuronal release and peripheral cellular contributions, thereby providing insight into disease-associated protein aggregation and clearance processes [25, 26]. Alterations in total, oligomeric, and phosphorylated forms of serum α-syn have been linked to PD progression and severity, supporting its potential diagnostic and prognostic value [27, 28]. This peripheral biomarker approach may be particularly relevant in toxin-induced models such as PQ exposure, where blood–brain barrier disruption could facilitate movement of neuronal proteins into systemic circulation [29].

Experimental rodent models, especially Wistar rats, are widely employed in studies of PQ-induced neurodegeneration because of their well-characterized neurobiology and predictable behavioural responses [30]. PQ administration in rodents has been associated with neuronal loss within the SNpc, behavioural impairments, and molecular alterations linked to Parkinsonian pathology [13]. Evaluating structural alterations within the SNpc across developmental stages may provide insight into how aging interacts with environmental toxicants to influence neurodegenerative processes. In parallel, neurobehavioural assessments provide functional indices of toxin-induced neurological impairment. Increasing age may further exacerbate PQ-induced neuronal injury through reduced cellular resilience and impaired reparative capacity [31]. It is imperative to note that rodent models of PQ exposure are primarily used to investigate mechanisms of dopaminergic neurotoxicity rather than to fully recapitulate idiopathic PD. These models are therefore better classified as models of toxin-induced Parkinsonism. Understanding these age-related patterns is therefore important for identifying mechanisms underlying differential susceptibility to neurodegeneration.

Despite evidence that PQ causes oxidative stress, α-syn pathology, and dopaminergic injury in experimental models [19], several important questions remain unresolved. Most studies have focused predominantly on adult animals [11, 32–34], leaving uncertainty regarding susceptibility across developmental stages. In addition, reports on the effects of PQ on total α-syn expression remain inconsistent, and relatively few studies have assessed whether circulating α-syn levels reflect neuropathological changes in PQ-induced neurotoxicity. Similarly, post-exposure recovery across age groups has not been systematically characterized, leaving uncertainty regarding whether PQ-induced damage is reversible or persistent.

This study is anchored on the well-established role of biological aging as a key modulator of susceptibility to neurodegenerative processes [33]. This foundational evidence informed the choice of study design, in which juvenile, young-adult, and adult male Wistar rats were exposed to a standardized PQ paradigm to enable direct comparative assessment of age-dependent vulnerability. Rather than proposing age as an independent or novel determinant of Parkinsonian risk, this approach clarifies how developmental stage influences neurotoxic outcomes under controlled experimental conditions, thereby providing a structured framework for evaluating differential susceptibility across the lifespan.

Accordingly, the present study investigated age-related differences in susceptibility to PQ-induced neurotoxicity in juvenile, young-adult, and adult male Wistar rats. Neurobehavioural performance, serum and nigral α-syn concentrations, and histoarchitectural changes within the substantia nigra were evaluated during exposure and following a defined recovery period. The study hypothesized that PQ-induced neurobehavioural impairment and nigral degeneration would vary according to age.

## Methods

### Experimental animals and grouping

Sixty-three male Wistar rats were obtained from the animal facility of the College of Veterinary Medicine, Michael Okpara University of Agriculture, Umudike, Nigeria. Animals were categorized according to baseline age into three cohorts: juvenile, young adult, and adult (6–8 weeks, > 8–12 weeks, and > 12 weeks respectively) [35]. Group classification was based on initial age of experimental animals in order to preserve the consistency of the experiment. Age classification was intended to represent early post-weaning (juvenile), periadolescent/young adult, and mature adult stages rather than senescent aging.

Following a two-week acclimatization period, rats were assigned using a simple random allocation procedure into control (C), paraquat (PQ), and paraquat + recovery (PQ + R) groups (*n* = 7) within each age cohort. Both PQ and PQ + R group received paraquat dichloride (10 mg/kg, i.p.) twice weekly for three weeks; however, PQ + R group was allowed a two-month recovery period before sacrifice. The recovery duration was adopted based on reported persistence of PQ in the ventral midbrain tissue with a half-life of about a month [36], allowing evaluation of sub-acute recovery following cessation of exposure and to assess persistence of neurotoxic effects beyond the active dosing phase The control group received an equivalent dosage of normal saline (vehicle) intraperitoneally.

### Animal housing

Polypropylene cages were used to house rats under standard laboratory conditions typically 22 ± 2 °C and 12-hour light/dark cycle with constant supply of standard rat feed and water. Bedding was replaced thrice weekly and animal health checks were conducted daily.

### Induction of experimental parkinsonism

Paraquat dichloride (Sigma-Aldrich, USA) was diluted with sterile normal saline to achieve a working concentration suitable for dosing at 10 mg/kg body weight. PQ was administered twice weekly intraperitoneally over a period of three weeks [37] using fresh PQ solutions prepared on each administration day in order to maintain chemical stability and avoid degradation or contamination dynamics. Body weights were checked prior to each administration to ensure accurate dosing.

### Neurobehavioral assessments

Behavioral testing was conducted at three time points namely; Baseline (pre-exposure), a day after final PQ administration, and at the end of the recovery period (PQ + R groups only). All behavioural assessments were performed between 09:00 and 14:00 h in a controlled environment and test apparatus were cleaned between trials with 70% alcohol in order to remove olfactory cues.

**Fig. 1:**
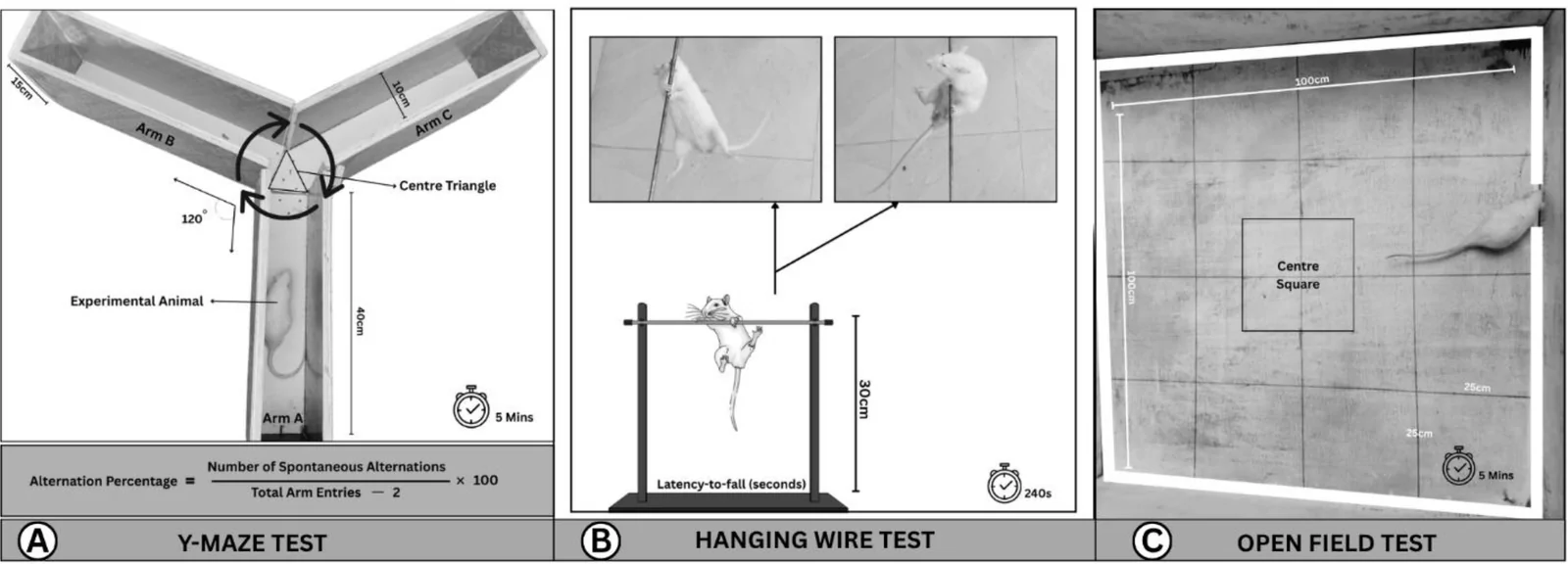
Neurobehavioral assessment apparatus and procedures

#### Open field test

Locomotor and exploratory behavior were evaluated in a square arena (100 × 100 × 40 cm) Fig. 1C. Each rat was placed on the central portion of the apparatus and allowed a 5-minute exploration time. Total line crossings (locomotor activity) and frequency of urination (stress-related response) were recorded. Higher line crossings was interpreted as improved exploratory activity while reduced number of urine pools was interpreted as improved anxiety-related behaviour [38].

#### Hanging wire test

Grip strength and neuromuscular endurance were assessed using a horizontal wire suspended 50 cm above base Fig. 1B. Latency-to-fall was recorded with a 240-second cut-off. Higher latency indicated better motor performance [39].

### Y-maze test

Spatial working memory was assessed using a three-arm Y-maze (40 × 10 × 15 cm) positioned at 120° angles Fig. 1A. Rats were allowed free exploration for five minutes. Spontaneous alternation percentage was calculated based on sequential arm entries without repetition. Higher alternation rates reflected improved working memory [40].

### Blood collection and serum preparation

Animals were euthanized under ketamine/xylazine anesthesia (80:10 mg/kg), and blood samples were collected via ocular puncture into plain sample tubes. The collected blood samples were allowed to clot at room temperature for 30 min. Thereafter, the samples were centrifuged at 3000 revolutions per minute (rpm) for 15 min at 4 °C, after which the serum was separated and stored at − 20 °C until biochemical analyses were performed.

### Brain harvesting

Brains were rapidly excised and weighed following decapitation in accordance with institutional guidelines and the American Veterinary Medical Association (AVMA) guidelines for animal euthanasia [41]. Relative brain weight was calculated as the ratio of brain weight to body weight. The left cerebral hemisphere was isolated, and the substantia nigra was microdissected on ice, homogenized in phosphate-buffered saline (pH 7.4), and centrifuged at 10,000 rpm for 15 min at 4 °C. The resulting supernatants were collected and stored at − 80 °C until biochemical analyses were performed. The right hemisphere, on the other hand, was prepared for cross-sectional histological examination by fixation in 10% neutral-buffered formalin.

### Histological procedures and light microscopy

Fixed tissues were processed routinely, embedded in paraffin wax, and coronally sectioned at 5 μm thickness using a rotary microtome. Tissue sections were subsequently stained with hematoxylin and eosin according to standard histological protocols [42], following which the SNpc was identified using established anatomical landmarks and characteristic histoarchitectural features. Photomicrographs were obtained using a calibrated brightfield microscope manufactured by Labomed Inc. at ×400 magnification under standardized illumination conditions. Image acquisition and analysis were performed using the PixelPro application (version 8.0) by an investigator blinded to group allocation.

### Quantification of alpha-synuclein

Alpha-synuclein levels in serum and substantia nigra homogenates were quantified using a commercial sandwich Enzyme Linked Immunosorbent Assay (ELISA) kit (Cell Signaling Technology, USA) according to the manufacturer’s protocol. Samples and standards were analyzed in duplicate (Absorbance: 450 nm; Concentrations expressed as ng/mL) while inter-assay variability was maintained below 10%.

### Statistical analysis

Statistical analyses were performed using GraphPad Prism (v10.0) and IBM SPSS Statistics (v25.0). Data are expressed as mean ± standard deviation (SD). Within-group comparisons of pre- and post-treatment behavioral scores were analyzed using paired t-tests. Treatment and age effects were evaluated using repeated-measures two-way (Analysis of Variance) ANOVA, while one-way ANOVA was applied for within-cohort comparisons where appropriate. Normality of data distribution was assessed using the Shapiro–Wilk test, while homogeneity of variances was evaluated using Levene’s test prior to parametric analyses. Significance was set at *p* ≤ 0.05.

### Ethical approval

All experimental procedures were reviewed and approved by the Animal Research Ethics Committee (AREC) of Nnamdi Azikiwe University (NAU); (Ref: NAU/AREC/2025/0059) on 12 May 2025. The study was conducted in accordance with the National Institutes of Health (NIH) guidelines for the care and use of laboratory animals [43].

## Results

### Neurobehavioral assessments

**Fig. 2:**
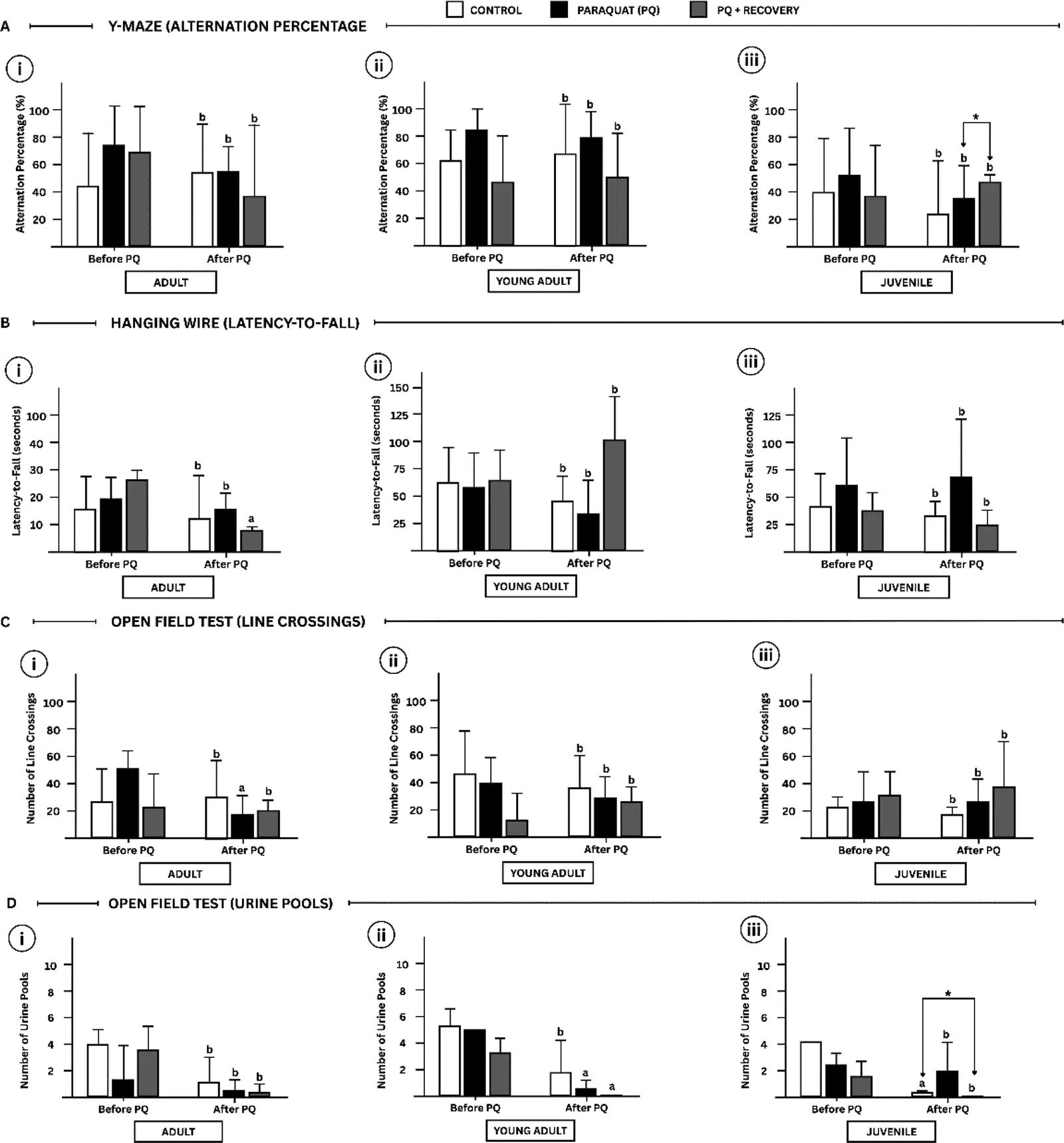
Neurobehavioural test outcomes—including alternation percentage (**A**), latency-to-fall (**B**), line crossings (**C**), and urine pools (**D**)—show mean performance before and after paraquat (PQ) administration across all age cohorts and experimental groups. a = significant “before PQ vs. after PQ” differences; b = non-significant “before PQ vs. after PQ” differences. *p* ≤ 0.05 considered statistically significant

#### Spontaneous alternation (%)

Spontaneous alternation performance did not differ significantly between pre- and post-treatment assessments across all experimental groups within each age category (*p* > 0.05). In addition, no significant age-by-treatment interaction was observed across cohorts (*p* > 0.05) Fig. 2A (i–iii).

#### Latency-to-fall (seconds)

Motor coordination assessed using the latency-to-fall test showed no significant differences between pre- and post-treatment measurements in most groups. A significant change was observed only in the adult PQ + R group (*p* = 0.05). Overall, no significant age-by-treatment interaction was detected across cohorts (*p* > 0.05) Fig. 2B (i–iii).

#### Line crossings (LC)

Locomotor activity, assessed by line-crossing frequency, remained comparable between pre- and post-test values in most experimental groups (*p* > 0.05). A significant reduction in locomotor activity was observed in adult rats exposed to paraquat (*p* = 0.02). However, no significant interaction between age and treatment was detected (*p* > 0.05) Fig. 2C (i–iii).

#### Urine pools (UP)

Urination patterns did not differ significantly among treatment groups in the juvenile and adult cohorts (*p* > 0.05). In contrast, the young-adult cohort showed significant treatment effects, with both the PQ group (*p* = 0.0010) and PQ + R group (*p* = 0.0016) differing significantly from controls. The recovery group also demonstrated altered urination frequency relative to controls (*p* = 0.05). No significant age-by-treatment interaction was observed (*p* > 0.05) Fig. 2D (i–iii).

#### Relative brain weight

**Table 1:** Relative brain weight across age cohorts following paraquat exposure and recovery

Age Cohort	Relative Weight (Mean ± SD)			
	**Control** (*n* = 7)	**PQ** (*n* = 7)	**PQ + R** (*n* = 7)	*p*-value
Adult	0.72 ± 0.09	0.74 ± 0.10	0.84 ± 0.17	0.196
Young Adult	0.79 ± 0.07	0.90 ± 0.05	0.88 ± 0.07	0.168
Juvenile	0.88 ± 0.07	0.89 ± 0.09	0.93 ± 0.05	0.645

Data are presented as mean ± standard deviation (SD). Relative brain weight was calculated as the ratio of organ weight to body weight (expressed as a percentage). *p* ≤ 0.05 considered statistically significant

Relative brain weight did not differ significantly among control, PQ, and PQ + R groups within the adult (*p* = 0.196, R² = 0.419), young-adult (*p* = 0.168, R² = 0.449), or juvenile (*p* = 0.645, R² = 0.136) cohorts (Table 1). These findings indicate that paraquat exposure at the administered dose did not produce measurable changes in gross brain mass across age groups.

### Serum alpha-synuclein levels

**Fig. 3:**
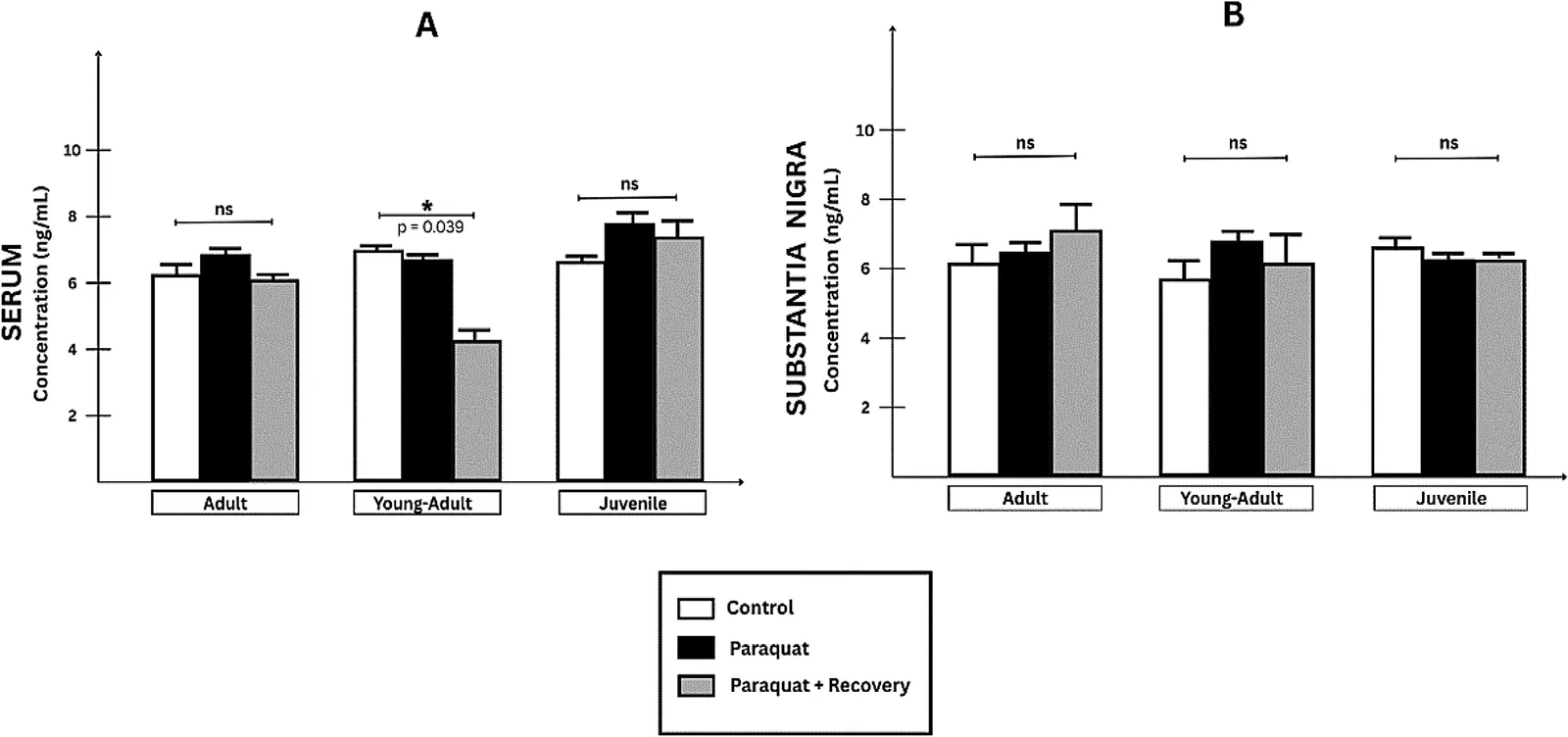
Alpha-synuclein concentrations in the serum (**A**) and substantia nigra (**B**) following paraquat administration. *p* ≤ 0.05 considered statistically significant. ns = not significant

Serum α-synuclein concentrations were comparable among treatment groups in the adult and juvenile cohorts (*p* > 0.05). In contrast, a significant difference was observed in the young-adult cohort (*p* = 0.039), where post hoc analysis revealed reduced serum α-syn levels in the PQ + R group compared with controls (*p* = 0.047) Fig. 3A.

### Substantia nigra alpha-synuclein levels

No significant differences in α-syn concentrations within the substantia nigra were observed among control, PQ, and PQ + R groups across all age categories (*p* > 0.05) Fig. 3B.

### Histological assessment of the substantia nigra

**Fig. 4:**
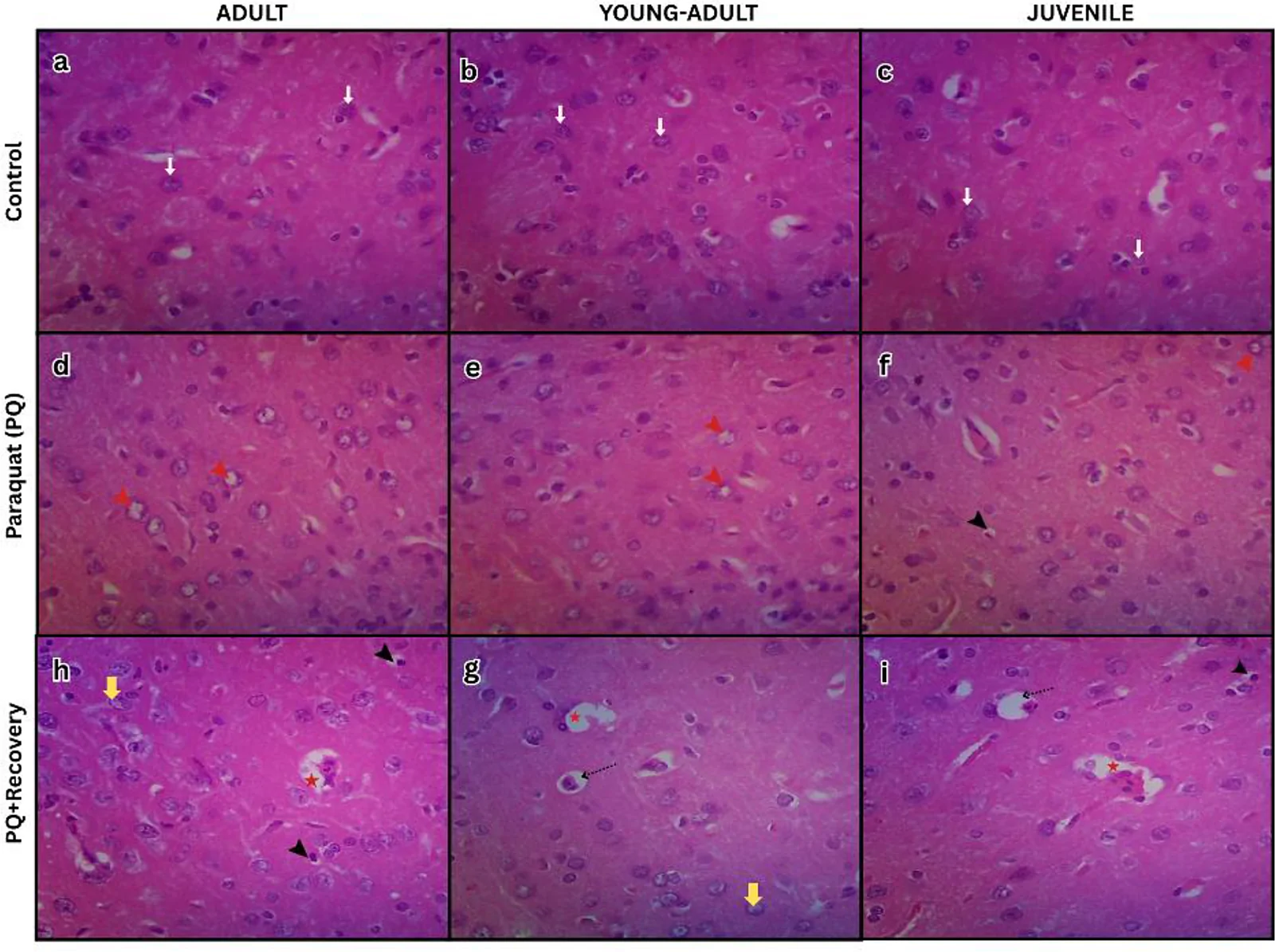
Photomicrograph of substantia nigra histoarchitecture across all groups (control, paraquat (PQ), and PQ+Recovery (PQ + R)) in each age cohort. (Plates a-c) represent control groups; (plates d-f) represent paraquat groups while (plates g-i) represent PQ + R groups [all across the adult, young-adult and juvenile cohort]

Histological examination of the substantia nigra pars compacta revealed distinct group-dependent cytoarchitectural changes. In control animals across all age categories, dopaminergic neurons displayed preserved architecture with intact cell bodies, minimal vacuolation, and well-organized tissue structure Fig. 4; Plates a–c. Following PQ exposure, morphological alterations were observed, particularly in adult animals, including cytoplasmic rarefaction and cellular shrinkage (red arrowhead), as well as nuclear pyknosis (black arrowhead). Similar features were also evident in the young-adult and juvenile cohorts, although they appeared less prominent Fig. 4; Plates d–f. In the PQ + R groups, residual histopathological changes persisted, including cytoplasmic clearing (yellow arrow), pericellular vacuolation (red star/broken arrow), and nuclear pyknosis (black arrowhead). Nevertheless, the overall tissue architecture appeared improved relative to the PQ-only groups, suggesting partial structural recovery following withdrawal of PQ exposure Fig. 4; Plates g–i. The descriptive nature of the histological assessment precludes definitive conclusions regarding the extent of neuronal loss or recovery.

## Discussion

The present study investigated age-dependent susceptibility to PQ-induced neurotoxicity in male Wistar rats across different developmental stages, with an additional recovery arm to evaluate persistence of functional and structural alterations following exposure cessation. Overall, the findings suggest that biological age modulates vulnerability to PQ-induced neurotoxic effects, with adult animals exhibiting comparatively greater alterations.

Adult rats exhibited greater vulnerability to PQ-induced structural and functional alterations within the nigrostriatal system, as evidenced by more pronounced histoarchitectural disruption and reduced locomotor activity. In contrast, juvenile and young-adult animals showed relatively mild changes, suggesting partial developmental resilience to oxidative neurotoxic insult at earlier life stages. Interestingly, younger cohorts demonstrated reduced urination frequency, which may represent early autonomic involvement preceding overt neuronal loss. These findings suggest that PQ toxicity manifests along a continuum, with structural damage and functional impairment becoming more evident as the nigrostriatal system matures.

The age-related vulnerability observed here is consistent with previous experimental studies linking PQ exposure to dopaminergic degeneration and motor deficits [32, 34, 44–47]. For example, Fahim et al. [48] used a similar dosing protocol and reported reduced locomotor activity; thus, supporting our adult cohort findings. However, Widdowson et al. [49] observed minimal effects at lower oral doses, underscoring the importance of exposure route and cumulative burden. Likewise, Thiruchelvam et al. [50] documented progressive dopaminergic deficits in older animals treated with PQ or PQ+maneb, further supporting the role of aging as a determinant of neurotoxic outcome. Furthermore, recent evidence supports the broader reproducibility of PQ-induced motor dysfunction across rodent models. In a systematic review and meta-analysis, PQ exposure was associated with significant substantia nigra pars compacta (SNpc) neuronal loss and measurable motor deficits across multiple behavioural paradigms including the open field, rotarod, rearing, and gait analyses [51]. Importantly, the authors reported that behavioural deficits were more likely to occur in models exhibiting substantial SNpc neuronal loss, suggesting a relationship between structural degeneration and motor impairment. Although the present study demonstrated relatively modest behavioural changes, particularly in adult animals, our findings are broadly consistent with the overall direction of evidence indicating that PQ can induce both nigrostriatal pathology and motor dysfunction.

The modest behavioural alterations observed, particularly the reduction in locomotor activity among adult animals, suggest that functional impairment may emerge before extensive neuronal loss becomes detectable. This dissociation may reflect compensatory dopaminergic mechanisms or threshold effects within the nigrostriatal pathway. The more pronounced structural alteration in the nigral cytoarchitecture observed in adults may be attributable to age-related declines in antioxidant defenses and mitochondrial efficiency [52], a mechanism consistent with previous reports of PQ-induced dopaminergic neurotoxicity [11, 34, 48, 53, 54]. However, the neurotoxic effects of PQ remain variable across studies. For example, Smeyne et al. [55] and Breckenridge et al. [56] reported limited or inconsistent evidence of dopaminergic neuronal loss following PQ exposure, despite demonstrable brain accumulation of the toxicant. Breckenridge et al. [56] further observed no significant alterations in striatal dopamine concentrations or activation of microglia and astrocytes in C57BL/6J mice. Such discrepancies likely reflect differences in experimental design, including species, strain, exposure route, dose, treatment duration, and outcome measures. PQ likely drives the behavioral deficits via oxidative stress, neuroinflammation, and disrupted dopaminergic signalling. Chronic PQ exposure has been shown to lower striatal dopamine and TH‑positive neurons, suppress antioxidant enzymes (SOD, GSH), and increase lipid peroxidation [57]. Similarly, PQ‑treated mice exhibited impaired motor performance with dopaminergic neuronal loss, elevated α‑syn, altered lipid metabolism, and raised systemic and central inflammatory cytokines [58]. Although most of these markers were not measured in the present study, they plausibly underlie the adult locomotor decline and nigral changes.Importantly, the absence of major behavioral deficits in younger rats does not necessarily indicate resistance, but rather suggests the presence of compensatory mechanisms. Stable spontaneous alternation performance implies preserved hippocampal function, while intact locomotion suggests temporary maintenance of dopaminergic transmission. Over time, however, such subclinical changes may accumulate, particularly as antioxidant defenses decline with age [55]. This observation parallels human epidemiological patterns in which advancing age is a key risk factor for dopaminergic neurodegeneration. Although partial cytoarchitectural recovery was noted after two months, persistent vacuolation in older rats suggests incomplete repair, consistent with slower regenerative processes in aging tissue [59].

The lack of significant differences in relative brain weight further suggests that PQ toxicity at this dosage produces subtle cellular injury rather than gross atrophy. As noted previously, neurodegenerative processes often precede measurable anatomical changes [60], limiting the sensitivity of whole-brain weight measurements in toxicological assessment.

### Alpha-synuclein levels in the serum and nigral tissue

Despite histological evidence of nigral structural alterations, total α-syn levels within the substantia nigra did not differ significantly across groups. This finding should be interpreted cautiously because alterations in α-syn biology do not necessarily manifest as changes in total protein concentration. Experimental evidence suggests that PQ exposure may preferentially influence α-syn aggregation, conformational state, cellular localization, and post-translational modification rather than overall abundance [31, 61]. Consistent with this, PQ-induced oxidative stress has been shown to enhance α-syn-mediated membrane dysfunction and cellular vulnerability in dopaminergic cell models, particularly when combined with dopamine-mediated oxidative stress [62]. Furthermore, structural and functional neuronal disturbances may emerge before measurable changes in total α-syn expression. Studies of early-stage toxin-induced Parkinsonism have demonstrated that neuronal degeneration, ultrastructural abnormalities, and behavioural impairments can occur despite relatively preserved levels of molecular markers, reflecting compensatory mechanisms, functional deficits, or early pathogenic processes that precede overt protein accumulation [61]. Therefore, unchanged total nigral α-syn levels do not exclude the possibility that pathogenic α-syn species were altered in the present model.

Our findings are consistent with previous reports indicating that PQ-induced neurotoxicity does not necessarily involve increased total α-syn expression [63, 64]. Similarly, Mitra et al. [65] demonstrated region-specific effects of PQ on α-syn expression, reporting increased immunoreactivity in the hippocampus and frontal cortex but reduced expression in the substantia nigra. Such observations suggest that PQ-induced alterations in α-syn may vary substantially across brain regions and may not follow a uniform pattern of accumulation throughout the central nervous system. Regional differences in α-syn regulation may therefore contribute to the occurrence of substantial nigral structural injury in the absence of detectable increases in total nigral α-syn concentration. In addition, induction of molecular chaperones such as HSP70 may limit excessive protein accumulation despite ongoing cellular stress [63].

Age-dependent differences were more apparent in serum α-syn levels, particularly the reduction observed in young adults after recovery. Such variation may reflect differences in protein turnover, oxidative stress handling, and clearance pathways [27, 29, 66, 67]. Because peripheral α-syn predominantly originates from erythrocytes and platelets [68–70], fluctuations in blood cell dynamics may substantially influence measured serum concentrations independent of central nervous system pathology.

Clinical studies have similarly reported inconsistent findings regarding circulating α-syn. Numerous ELISA-based investigations have found no significant differences between controls and patients with PD [71–80], whereas others have reported either reduced levels [81, 82] or elevated concentrations [83–86]. Alternative detection platforms, including electro-analytical methods and immunomagnetic reduction, have also yielded conflicting results [87–93]. Collectively, these findings suggest that serum α-syn lacks sufficient consistency to serve as a standalone biomarker of nigrostriatal pathology.

The absence of significant α-syn accumulation in the present study is consistent with observations that toxin-induced models do not universally reproduce classical α-syn pathology, as highlighted by Dovonou et al. [30]. Instead, PQ is thought to exert neurotoxicity primarily through reactive oxygen species generation and Nicotinamide Adenine Dinucleotide Phosphate (NADPH) oxidase activation [94], mechanisms capable of altering α-syn structure and function without necessarily increasing its overall abundance. Emerging evidence further indicates that oxidative stress, neuroinflammation, mitochondrial dysfunction, and α-syn pathology interact in a complex and bidirectional manner. For example, PQ-induced motor deficits have been associated with increased inflammatory lipid mediators, elevated cytokine production, and altered α-syn expression [58], while Akhter et al. [95] reported concurrent up-regulation of α-syn, IL-1β, IL-6, and TNF-α transcripts following PQ exposure. Together, these findings support the concept that PQ-induced neurotoxicity involves interconnected oxidative and inflammatory pathways that may influence α-syn homeostasis. However, because the present study quantified only total α-syn levels and did not assess oxidative stress or inflammatory markers, direct mechanistic conclusions regarding these interactions cannot be drawn.

Although environmental toxicants such as PQ have been implicated as potential risk factors for Parkinsonian syndromes, the extent to which toxin-induced models reproduce the pathobiology of idiopathic Parkinson’s disease remains debated. Toxin-based models are useful for exploring mechanisms of oxidative stress, mitochondrial dysfunction, and dopaminergic vulnerability; however, they do not consistently replicate key features of idiopathic Parkinson’s disease, including progressive Lewy body formation, widespread α-syn pathology, and the prolonged course of neuronal degeneration observed in humans [96, 97]. Therefore, findings derived from PQ models should be interpreted primarily within the context of environmentally induced Parkinsonism rather than as complete representations of idiopathic Parkinson’s disease. Nevertheless, these models remain useful for investigating specific pathogenic pathways that may contribute to neurodegeneration and may help unveil how environmental exposures interact with biological susceptibility factors [97].

### Limitations of the study

Several limitations should be acknowledged. First, the study did not include tyrosine hydroxylase (TH) immunohistochemistry, which would have provided direct confirmation of dopaminergic neuronal loss. Second, histological analysis was limited to hematoxylin and eosin (H&E) staining, which does not permit specific identification of dopaminergic neurons or quantitative assessment of nigrostriatal pathway integrity. Third, behavioral assessment was restricted to the open field, Y-maze, and hanging wire tests, which do not comprehensively capture the spectrum of Parkinsonian motor deficits, including bradykinesia, rigidity, postural instability, and gait abnormalities. Fourth, mechanistic biomarkers of oxidative stress (e.g., malondialdehyde [MDA], superoxide dismutase [SOD], catalase [CAT], and reduced glutathione [GSH]) and neuroinflammation (e.g., TNF-α, IL-1β, IL-6, GFAP, and Iba-1) were not evaluated, thereby limiting mechanistic interpretation of the observed neurotoxicity. Fifth, the relatively small sample size (*n* = 7 per group) may have limited statistical power for detecting subtle behavioral and biochemical differences. Finally, although adult animals exhibited greater susceptibility than juvenile and young-adult cohorts, the absence of a senescent animal group limits extrapolation to the late-life neurodegeneration characteristic of idiopathic Parkinson’s disease.

### Future directions

Future studies should incorporate TH immunohistochemistry, stereological cell counting, and direct neurochemical assessment of the nigrostriatal pathway to strengthen evidence of dopaminergic dysfunction. Future studies should also incorporate a broader battery of behavioural assessments that are specific to Parkinsonian motor dysfunction, including rotarod, pole and cylinder tests, gait analysis, and assessments of postural stability and rigidity in order to provide a more comprehensive characterization of motor impairment. Given that the present study quantified only total α-syn, future work should also investigate pathological α-syn species, including oligomeric and phosphorylated forms, using aggregate-specific methodologies. These species may represent more sensitive indicators of early neurodegenerative processes and may possess greater diagnostic and translational utility than measurements of total α-syn alone. In addition, integrating age-dependent analyses of mitochondrial function, antioxidant capacity, and proteostatic mechanisms may provide further insight into the biological basis of differential susceptibility to PQ-induced neurotoxicity. Considering the variability of serum α-syn findings reported across both experimental and clinical studies, multi-marker approaches incorporating Neurofilament Light Chain (NfL), Protein Deglycase DJ-1 (DJ-1), inflammatory mediators, and omics-based strategies may offer greater translational utility. Finally, extended longitudinal studies incorporating additional post-exposure time points would help determine whether the subtle alterations observed in younger animals ultimately progress to persistent neurodegeneration with advancing age.

## Data Availability

All data generated or analysed during this study are included in this published article.

## Abbreviations

6-OHDA: 6-Hydroxydopamine
α-syn: Alpha-synuclein
ANOVA: Analysis of variance
AREC: Animal research ethics committee
AVMA: American veterinary medical association
BMI: Body mass index
CAT: Catalase
cm: Centimeter
CSF: Cerebrospinal fluid
DJ-1: Parkinson disease protein 7 (PARK7)
ELISA: Enzyme-linked immunosorbent assay
GFAP: Glial fibrillary acidic protein
GSH: Reduced glutathione
HSP70: Heat shock protein 70
Iba-1: Ionized calcium-binding adapter molecule 1
IBM: International business machines
IL-1β: Interleukin-1 beta
IL-6: Interleukin-6
i.p.: Intraperitoneal
kg: Kilogram
LC: Line crossings
MDA: Malondialdehyde
mg/kg: Milligram per kilogram
MPTP: 1-Methyl-4-phenyl-1,2,3,6-tetrahydropyridine
µm: Micrometer
NADPH: Nicotinamide adenine dinucleotide phosphate (reduced form)
NAU: Nnamdi Azikiwe university
NfL: Neurofilament light chain
ng/mL: Nanogram per milliliter
NIH: National institutes of health
p: Probability value
PD: Parkinson’s disease
PixelPro: PixelPro image analysis software
PQ: Paraquat
PQ + R: Paraquat plus recovery
R²: Coefficient of determination
ROS: Reactive oxygen species
rpm: Revolutions per minute
SD: Standard deviation
SNCA: Synuclein alpha gene
SNpc: Substantia nigra pars compacta
SOD: Superoxide dismutase
SPSS: Statistical package for the social sciences
TH: Tyrosine hydroxylase
TNF-α: Tumor necrosis factor-alpha
UP: Urine pools
USA: United States of America
×400: Four hundred times magnification
°C: Degree celsius
